# Well-being as a performance pillar: a holistic approach for monitoring tennis players

**DOI:** 10.3389/fspor.2023.1259821

**Published:** 2023-09-18

**Authors:** Marie-Florine Michel, Olivier Girard, Vincent Guillard, Cyril Brechbuhl

**Affiliations:** ^1^ Faculty of Sports Science, Aix-Marseille University, Marseille, France; ^2^ French Tennis Federation, Stade Roland-Garros, Paris, France; ^3^ School of Human Sciences (Exercise and Sport Science), The University of Western Australia, Crawley, WA, Australia

**Keywords:** training monitoring, well-being, prevention, performance optimization, tennis

## Abstract

This perspective article aims to discuss the usefulness of tools that can assist tennis professionals effectively manage the well-being of their players. This includes identifying and monitoring meaningful metrics (i.e., training load, training intensity, heart rate variability), as well as careful planning of training and competition schedules with appropriate recovery periods. The use of innovative training methods (i.e., repeated-sprint training in hypoxia and heat training), and proper dietary practices, along with biometric assessment for young players, represents should be considered. Adopting a holistic approach to decision-making about training and competition, balancing both health and performance considerations, is crucial for tennis players and their support teams. More research is needed to refine best practices for enhancing tennis performance while prioritizing the well-being of players.

## Introduction

In recent years, we have witnessed a significant rise in injuries among the top tennis players competing in Grand Slam tournaments (1). This is concerning, as sustaining an injury can have serious implications for a player’s career, potentially leading to a decline in rankings and even endangering their presence on the tour. Injury not only affects top-level adult tennis players but also elite junior tennis players. Approximately one out of every eight elite juniors tennis players encounters health-related issues weekly, encompassing overuse injury, medical illness, and acute injuries (2). Moreover, in a separate study, 41% of these young athletes reported experiencing injuries within a year (3). Considering the link between good health and optimal performance, perhaps prioritizing players’ well-being may hold the key to unlocking peak performance.

In the August 2023 rankings, there were three male players and one female player aged 21 or younger in the Association of Tennis Professionals and the Women's Tennis Association Top 10, compared to none a decade ago. Professionalization is beginning at an increasingly young age in tennis. Hence, our approach extends to players aged 10 and above, in line with the growing trend of early professionalization in tennis. The influence of the 10,000 h rule and the 10-year rule in highly coordinated sports further shapes our approach (4). Professional tennis players face a grueling and often largely unpredictable schedule of matches, training, and travel throughout the year. The players’ competition schedule is determined by tournament play, which dictates the periodization of their calendars. Tournament opportunities arise on a weekly basis at the highest level, resulting in approximately 30 weeks of competition per year, with only about 20 weeks dedicated to training and recovery (5). To accumulate ranking points and gain prestige and recognition, players must maintain peak performance, while also considering their well-being. General (i.e., physiological and psychological) well-being in this context can be understood as a combination of happiness derived from pleasurable experiences and a sense of living up to one's potential and “true self” (6). However, the demanding nature of the sport and the high frequency of tournaments can take a toll on their physical condition and increase the risk of injuries. Indeed, studies revealed that injured athletes often experience lower self-esteem and higher levels of depression and anxiety compared to their uninjured counterparts (7–9). Therefore, meticulous planning of the training program and a comprehensive approach to player well-being become essential factors in maximizing performance and minimizing injury risks.

At the core of player monitoring practice lie two fundamental constructs: performance improvement and injury prevention. Although distinct, these constructs are closely linked, as injuries and subsequent inability to train can significantly impact player performance (10). Recognizing this interconnection, adopting a comprehensive approach to training and player well-being becomes paramount. By effectively implementing systematic monitoring techniques, optimizing training approaches, and embracing innovative methods, junior tennis players can not only enhance performance but also safeguard players’ health and minimize the risk of injuries. By integrating these elements into a cohesive player management approach, coaches and support teams can collaborate to unlock the true potential of their young players, fostering not only improved performance but also long-term general health and success on the court.

To ensure a comprehensive approach to training and performance, tennis professionals must implement a systematic monitoring plan that ultimately identifies key metrics. This will help them maintain a balanced approach to training and competing while managing the players’ health and competition preparedness (11). Often, the focus is on performance improvement, which may overlook potential general health consequences. To gauge the player's day-to-day readiness, various tools for assessing training responsiveness and general fatigue can be considered. These include monitoring factors such as training volume, duration, intensity, load, and/or type, along with skill repetitions, maximal oxygen uptake, rating of perceived exertion (RPE), heart rate variability (HRV), functional movement screen (FMS™), and peak height velocity (PHV). Additionally, such monitoring encompasses aspects related to recovery periods and techniques, sleep quantity and quality, and nutrition. This approach allows for a better understanding of the relationship between the collected data and the evaluated performance construct (12). Identifying key physical determinants of performance enables coaches and sports scientists to track individual players’ fitness responses through specific testing during the season, optimizing their on-court physical qualities. Although studies indicate that both over-training or under-training can increase injury risk (13), appropriate training may have a protective effect against injury (14).

In the following sections, we will delve into various aspects of optimizing training and performance in tennis players. This will encompass strategic monitoring of training load and intensity, biometric assessment follow up, early fatigue detection, and the potential benefits of innovative training techniques as tournaments approach.

## Training monitoring and optimization

Training blocks are an integral part of the development of junior elite players. Typically, these players adhere to tailored competition schedules that align with specific competitive priorities, such as ITF junior events. These training blocks are strategically placed between tournament clusters, enabling coaches to allocate dedicated tennis sessions for refining specific goals including technical, tactical, physical, or mental aspects (15). This approach emphasizes the individualized nature of junior player development and underscores the importance of employing various tools to facilitate their progress.

By utilizing load monitoring techniques, coaches can make informed decisions to fine-tune training programs. A growing number of studies have measured training load in arbitrary units (AU) on a weekly basis using the RPE in developing tennis players (5, 16). Gomes et al. (17) demonstrated that the session RPE method effectively serves as an indicator of training and competition loads in professional tennis players. This method helps coaches compare planned and perceived loads and plan future training based on players’ responses. However, this method acknowledges the potential for athletes to perceive the effort required as greater than anticipated in the prescribed easy sessions (18). Care should be taken to expose players to match-like physical intensities and training loads during preparatory training blocks, as training load is often increased during preparation periods compared to competition (19). In tennis, Perri et al. (5) found that loads tend to be higher during focused training periods than during competitions for players aged between 12 and 18, due to higher weekly training volumes. Effective monitoring allows for achieving desired physiological adaptations, as determined by the physical preparation staff, in the absence of competitive requirements. Increased training volume, in particular, promotes the development of the crucial aerobic base in tennis. Nonetheless, Gomes et al. (20) demonstrated that the load during official matches (881.3 ± 375.1 AU) is approximately 60% higher than that observed during training sessions (551.9 ± 183.0 AU) in 12 professional tennis players (age: 18.5 ± 0.4 years). Moreover, similar RPE scores were observed during official and simulated tennis matches. Overall, match RPEs typically range from 5 to 8 (out of 10), with loads ranging from 370 to 1,078 AU (5, 20, 21). Through close monitoring of training loads and player responses, using techniques like the session RPE method, coaches can make informed decisions to optimize training programs. This involves considering the variation in load between official matches and training sessions.

Within the context of tennis training, intensity is a crucial aspect that impacts players’ physiological adaptations. A proposed model with three intensity zones, based on the heart rate (HR) response associated with reproducible metabolic demarcation points [i.e., ventilatory thresholds (VT)], allows for the examination of physiological strain during tennis training. This HR-based model defines the following distribution: Zone 1 (low intensity, ≤VT1), Zone 2 (moderate intensity, >VT1 and <VT2), and Zone 3 (high intensity, ≥VT2). Researchers have found that a ‘pyramidal’ intensity distribution model is commonly followed in young elite tennis players (age: 13.8 ± 1.0 years), allocating approximately 75% of training time to low-intensity activities, 20% to moderate-intensity exercises, and 5% to high-intensity drills (16). This model promotes the development of the aerobic base, which plays a pivotal role in tennis performance. Careful management of the intensity load, considering both the HR zones and the distribution model, is essential to achieve desired physiological improvements and enhance overall player performance on the court. By aligning training intensity zones with the HR response, coaches and sports scientists can create more effective and individualized training programs, ensuring players reach their optimal fitness levels.

As tournaments approach, it is crucial to reduce the training volume and load while maintaining the “specificity” of on-court training exposure. With the modern tennis game becoming increasingly dynamic and tournament schedules more demanding, adapted strategies to improve or maintain physical fitness are needed (22). In this context, innovative training methods such as repeated-sprint training in hypoxia (RSH) may represent an effective short-term intervention for tennis players to optimize their sport-specific fitness levels, particularly during consecutive tournaments. Brechbuhl et al. (23) propose programming this intervention 2–3 times per season to develop or maintain the players’ aerobic capacity and ball accuracy. By incorporating five RSH sessions over a 12-day preparation period, beneficial adaptations can be triggered for both sprinting and tennis-specific performance without additional fatigue (23). By strategically implementing RSH in their training regimen, tennis players can enhance their physical capabilities and maximize fitness, thereby improving their performance during demanding tournament schedules.

Besides hypoxic training, heat-based methods can also improve players’ performance and acclimatize them to high-temperature conditions. Recommendations for heat acclimatization include engaging in repeated training sessions lasting at least 60 min per day, which induce marked increase in body core and skin temperatures, as well as stimulate sweating (24). Ideally, players should also train in the same environment as the competition venue. Early adaptations, constituting up to 75% of physiological adjustments and performance improvements, typically manifest within the first 4–6 days of repeated heat exposure (25). Maximal adaptation is generally achieved after a period of 10–14 days of heat exposure (26).

To accurately assess players’ progress and performance, the Test to Exhaustion Specific to Tennis (TEST) is commonly used (e.g., French Tennis Federation). This on-court procedure includes elements of the game such as ball hitting, lateral movements, and intermittent exercise, simulating tennis dynamics. This test evaluates players’ technical and physiological changes at specific intensity thresholds, helping coaches and sports scientists identify individual strengths and weaknesses for personalized training programs (27). The TEST allows for various training approaches, depending on the objectives, whether they are technical-focused (Zones 1 and 2) or energy-focused (Zone 3), always emphasizing the precision and speed of strikes with appropriate feedback (28). For instance, in Zone 1, the objective is to maintain the workout as long as possible at VT1, with 2 or 3 series of continuous play lasting 5–10 min each, followed by 3 min of passive recovery period between series (28). On the other hand, for Zone 3, shorter striking intervals (15 s–1 min) are preferred, with passive rest periods (15–30 s) in between. During the TEST, expert players aim to maintain intense hitting 70% of shots on the designated target, while lower-level players have a tolerance of up to 50%. Regularly conducting this specific test enables continuous evaluation of players’ progress and the effectiveness of training interventions, ensuring optimal resource utilization and maximizing training efficiency.

## Biometric assessment follow up

As players reach ages around 14, a careful and individualized approach to training becomes even more crucial, especially from a health perspective. During this stage of development, players undergo significant physical and physiological changes [i.e., peak height velocity (PHV)], making it essential to adapt training strategies to their evolving specific needs and capabilities [i.e., functional movement screen (FMS™)]. Understanding their unique growth and physiological characteristics becomes paramount in optimizing their potential for overall well-being and reducing susceptibility to overuse injuries, such as Osgood-Schlatter disease and Little Leaguer's elbow. This approach not only safeguards their development but also establishes a foundation for sustained athletic careers.

During the pubertal growth spurt, a significant transition occurs, increasing utilization of anaerobic pathways that produce metabolites leading to muscular fatigue (29). As a result, it becomes crucial to implement a high volume of low-intensity aerobic training to delay fatigue, facilitate recovery, and optimize technical training (30). By utilizing objective measurements of anthropometric variables (31), players can receive training tailored to their biologic status instead of their chronological age (32), using the pivotal marker of maturation being PHV. This player-centered approach emphasizes individualization and overall development, fostering a sense of well-being and providing long-term gains. Understanding the timing of the PHV enables coaches and sports scientists to design training regimens that emphasize specific aerobic exercises, which are particularly crucial during this critical phase of development. Moreover, coaches must consider maturation differences, as tennis players with a more advanced biological age tend to possess superior speed, agility, and explosive power qualities compared to their younger counterparts (33). To further enhance the performance in young tennis players during the pre-puberty and early puberty phases, additional studies should determine the most effective training approaches and content tailored to each maturity stage, as well as to examine the exact impact of PHV on injury risk.

The FMS™ is a popular movement screen that assesses fundamental movements, emphasizing the balance between stability and mobility in a proximal to distal sequence (34). It comprises seven individual movement patterns, each qualitatively assessed and scored based on compensatory movements or the presence of pain during the pattern. Several studies have explored the FMS™, investigating normative values, differences in scores based on sex, body composition, and skill level, as well as its reliability, validity, and potential association with injury (34). A significant area of study revolves around the relationship between FMS™ scores and the musculoskeletal injury risk. However, the findings have been mixed, with some studies reported a significant higher likelihood of an injury with low FMS™ scores (35), while others have not found strong predictive value (36). Although the FMS™ lacks strong evidence as a robust injury prediction tool, it can still be utilized to enhance individual workout or training plans and potentially improve performance. For example, individuals with poor FMS™ scores tend to exhibit compensatory movement patterns during regular activities. If left unchecked, these patterns may reinforce sub-optimal movement mechanics and increase the likelihood of future injuries (37).

## Early fatigue detection and recovery techniques

In conjunction with monitoring training intensity and employing period-specific training methods, it is essential to adopt tools that can routinely detect early signs of fatigue. One such promising tool is heart rate variability (HRV), which can be used to monitor fatigue states (38). HRV should be integrated into a comprehensive monitoring program as it reflects overall stress. Higher HRV is associated with improved recovery and readiness for training, while intense exercise acutely decreases vagal-related HRV indices for 24–48 h (39). Therefore, the integration of HRV monitoring within a holistic program may allow for the adjustment of training plans and the identification of optimal training load and recovery strategies for each player. HRV assessment encompasses various techniques such as electrocardiography, interbeat interval measurement, and photoplethysmography (40). Smartphone applications, connected to a heart rate monitor, have gained popularity for HRV monitoring due to their convenience and affordability, effectively addressing the practical needs of coaches and practitioners. Furthermore, commercial heart rate monitors have been shown to be a valid tool for HRV analysis during the resting state and incremental exercise (41). To implement this, player can perform HRV tests under identical conditions, either every morning before the breakfast, immediately upon waking up, or after a training session, match, and/or recovery period, even in the comfort of their homes. The test involves spending 5 min in a supine position followed by 5 min in a standing position, allowing for the measurement of HR, root-mean-square of successive R-R interval differences (RMSDD), and spectral power values. These data are then decomposed into low-frequency (LF) which reflects vagal modulation, and high-frequency (HF) bands, indicating both sympathetic and parasympathetic influences, after processing (42).

Both researchers and practitioners are increasingly focusing on improving the recovery capabilities of tennis players, with the aim of mitigating fatigue effects and expediting recovery processes (43). Various recovery techniques have been explored, including compressive clothing and temperature-based interventions such as cold-water immersion [between 5°C and 15°C (43)]. The latter method effectively reduces body (core, muscle and skin) temperature, inflammation, and heart rate, resulting in improved performance measures (i.e., endurance, strength, sprint, and jumps activities) by 2%–3% (43). Another technique involves the use of compressive clothing, which applies hydrostatic pressures to reduce muscle soreness, fatigue, and oedema, while enhancing the clearance of lactate and creatine kinase (43).

Nutrition also plays a pivotal role in optimizing recovery, overall health and well-being, and performance for tennis players. Proper dietary practices, including rehydration and consumption of a well-rounded diet with a variety of nutrient-rich foods, are essential for meeting the nutritional needs of players. Adequate intake of macronutrients, such as carbohydrates (6–10 g.kg^−1^.d^−1^) and proteins (∼1.6 g.kg^−1^.d^−1^), is crucial for replenishing energy stores and aiding in muscle recovery (43). A balanced diet brings about numerous benefits that become evident over the course of the season, inducing reduced injuries (44), improved recovery (45), and enhanced training capacity (46). These factors contribute to an overall well-being and performance progression throughout the season. Additionally, incorporating specific supplements into a player's nutrition plan can greatly contribute to optimal athletic performance and overall health and well-being (47). For instance, caffeine in doses of 3 mg.kg^−1^ can provide ergogenic benefits when taken before and/or during tennis match play, enhancing alertness and performance on the court (48). Furthermore, players should aim to consume 200 ml of electrolytes-containing fluid every 15 min in mild to moderate ambient temperatures (<27°C) (48), adjusting their intake based on their individual thirst levels. Moreover, when match play exceeds 2 h, players are advised to ingest 30–60 g.h^−1^ of carbohydrates to sustain energy levels and support their endurance during prolonged matches (48). By adopting these tennis-specific nutritional strategies, players can optimize their performance and overall well-being on the court.

Considering the paramount significance of sleep, it is recommended to adhere to the recommendations set forth by the National Sleep Foundation. These guidelines advocate for 8–10 h of nightly sleep for adolescents aged 13–18 (49). The importance of these guidelines is reinforced by Watson et al. (50), who underscore sleep's pivotal role in health and well-being, impacting physical development, emotional regulation, cognitive performance, and overall quality of life. Combining good sleep hygiene practices and mindfulness techniques has demonstrated the ability to enhance sleep quality, perceptual well-being, and mitigate tournament-related anxiety (51). Adequate sleep plays a critical role in athletic performance (50), particularly in sports like tennis that require speed, tactical strategy, and technical skill (52). Following sleep hygiene recommendations has even been linked to increased playing time and more shots taken (53). Long-term interventions aimed at extending sleep duration have resulted in significant improvements, including a 20 to 30% increase in serve accuracy (54). Prioritizing adequate sleep not only reduces the risk of injury and illness in athletes but also optimizes health and the potential to enhance performance through increased training participation. Coaches are therefore encouraged to implement sleep hygiene education and mindfulness techniques to reinforce healthy sleep behaviors and improve the performance of junior players.

## Where to now?

As we progress in optimizing training and performance for tennis players, several areas warrant further exploration and research. Firstly, there is a growing need for innovative training methods that can address the challenges posed by a grueling and unpredictable tournament schedule. Research on the effectiveness of RSH and heat-based training methods, and how these interventions may be combined, could provide valuable insights into their impact on players’ performance in challenging conditions. The choice of one of these two approaches will also depend on the current training phase, upcoming competitions, and individual athlete considerations. By investigating the optimal timing and frequency of these training interventions, coaches and sports scientists can develop more tailored training regimens that maximize players’ physical capabilities and improve their on-court performance during tournaments. Furthermore, while the use of biometric assessments shows promise in guiding personalized training strategies, more comprehensive studies are needed to establish its robustness as an injury prediction tool in tennis players. Further research can help validate the relationship between FMS™ scores and musculoskeletal injury risk and shed light on its potential benefits in preventing injuries and enhancing performance over the long term. Finally, the exploration of new technologies and methodologies, including artificial intelligence and machine learning [e.g., supervised learning (55)], for early fatigue detection and recovery is essential in providing timely interventions to optimize players’ readiness for training and competition. Advancements in HRV monitoring and other fatigue assessment techniques can help coaches and players fine-tune training plans and recovery strategies, ultimately contributing to better overall player management.

## Concluding remarks

Our comprehensive examination underscores the fundamental principles for optimizing training and ensuring the well-being of young tennis players. It emphasizes the importance of holistic player management, which involves the integration of systematic monitoring (i.e., training load, training intensity), innovative training methods (i.e., RSH, heat acclimatization), tailored biometric assessments (i.e., FMS™, PHV), early fatigue detection (i.e., HRV), effective recovery strategies (i.e., temperature-based interventions), attention to nutrition (i.e., well-rounded diet), and promoting healthy sleep hygiene (i.e., 8–10 h of nightly sleep). Continuous research and collaboration between sports scientists, coaches, and support teams will further refine these approaches and drive improvements in tennis performance and injury prevention.

## Data Availability

The original contributions presented in the study are included in the article/Supplementary Material, further inquiries can be directed to the corresponding author.

## References

[1] Sell K Hainline B Yorio M Kovacs M. Injury trend analysis from the US open tennis championships between 1994 and 2009. Br J Sports Med. (2014) 48(7):546–51. 10.1136/bjsports-2012-091175 22923462

[2] Pluim BM Loeffen FGJ Clarsen B Bahr R Verhagen EALM. A one-season prospective study of injuries and illness in elite junior tennis: injuries and illness in elite junior tennis. Scand J Med Sci Sports. (2016) 26(5):564–71. 10.1111/sms.12471 25944058

[3] Kovacs M Ellenbecker T Kibler Roetert EP Lubbers. Injury trends in American competitive junior tennis players. J Med Sci Tennis. (2014) 19:19–24.

[4] Issurin VB. Evidence-based prerequisites and precursors of athletic talent: a review. Sports Med. (2017) 47(10):1993–2010. 10.1007/s40279-017-0740-0 28493064

[5] Perri T Duffield R Murphy A Mabon T Reid M. Periodisation in professional tennis: a macro to micro analysis of load management strategies within a cluttered calendar. Int J Sports Sci Coach. (2022) 18(3):772–80. 10.1177/17479541221091087

[6] Giles S Fletcher D Arnold R Ashfield A Harrison J. Measuring well-being in sport performers: where are we now and how do we progress? Sports Med. (2020) 50(7):1255–70. 10.1007/s40279-020-01274-z 32103451 PMC7305091

[7] Chan CS Grossman HY. Psychological effects of running loss on consistent runners. Percept Mot Skills. (1988) 66(3):875–83. 10.2466/pms.1988.66.3.875 3405713

[8] McGowan RW Pierce EF Williams M Eastman NW. Athletic injury and self diminution. J Sports Med Phys Fitness. (1994) 34(3):299–304. 7830395

[9] Leddy MH Lambert MJ Ogles BM. Psychological consequences of athletic injury among high-level competitors. Res Q Exerc Sport. (1994) 65(4):347–54. 10.1080/02701367.1994.10607639 7886284

[10] Drew MK Raysmith BP Charlton PC. Injuries impair the chance of successful performance by sportspeople: a systematic review. Br J Sports Med. (2017) 51(16):1209–14. 10.1136/bjsports-2016-096731 28446456

[11] Soligard T Schwellnus M Alonso JM Bahr R Clarsen B Dijkstra HP How much is too much? (Part 1) international olympic committee consensus statement on load in sport and risk of injury. Br J Sports Med. (2016) 50(17):1030–41. 10.1136/bjsports-2016-096581 27535989

[12] West SW Clubb J Torres-Ronda L Howells D Leng E Vescovi JD More than a metric: how training load is used in elite sport for athlete management. Int J Sports Med. (2021) 42(04):300–6. 10.1055/a-1268-8791 33075832

[13] Gabbett TJ. The training—injury prevention paradox: should athletes be training smarter and harder? Br J Sports Med. (2016) 50(5):273–80. 10.1136/bjsports-2015-095788 26758673 PMC4789704

[14] Pluim BM Staal JB Windler GE Jayanthi N. Tennis injuries: occurrence, aetiology, and prevention. Br J Sports Med. (2006) 40(5):415–23. 10.1136/bjsm.2005.023184 16632572 PMC2577485

[15] Reid M Quinlan G Kearney S Jones D. Planning and periodization for the elite junior tennis player. Strength Cond J. (2009) 31(4):69–76. 10.1519/SSC.0b013e3181afc98d

[16] Michel MF Duboscq JM Ratel S Schmitt L Hidalgo A Brechbuhl C. Distribution of intensities and quantification of training load in young U15 elite tennis players. ITF Coach Sport Sci Rev. (2022) 30(88):4–9. 10.52383/itfcoaching.v30i88.329

[17] Gomes R Moreira A Lodo L Capitani C Aoki M. Ecological validity of session RPE method for quantifying internal training load in tennis. Int J Sports Sci Coach. (2015) 10(4):729–37. 10.1260/1747-9541.10.4.729

[18] Inoue A Dos Santos Bunn P Do Carmo EC Lattari E Da Silva EB. Internal training load perceived by athletes and planned by coaches: a systematic review and meta-analysis. Sports Med Open. (2022) 8(1):35. 10.1186/s40798-022-00420-3 35244801 PMC8897524

[19] Juhari F Ritchie D O’Connor F Pitchford N Weston M Thornton HR The quantification of within-week session intensity, duration, and intensity distribution across a season in Australian football using the session rating of perceived exertion method. Int J Sports Physiol Perform. (2018) 13(7):940–6. 10.1123/ijspp.2017-0626 29283733

[20] Murphy AP Duffield R Kellett A Reid M. A comparison of the perceptual and technical demands of tennis training, simulated match play, and competitive tournaments. Int J Sports Physiol Perform. (2016) 11(1):40–7. 10.1123/ijspp.2014-0464 25849156

[21] Coutts A Gomes R Viveiros de Castro LE Aoki M. Monitoring training loads in elite tennis. Rev Bras Cineantropometria E Desempenho Hum. (2010) 12(3):217–20. 10.5007/1980-0037.2010v12n3p217

[22] Reid M Schneiker K. Strength and conditioning in tennis: current research and practice. J Sci Med Sport. (2008) 11(3):248–56. 10.1016/j.jsams.2007.05.002 17597004

[23] Brechbuhl C Brocherie F Willis SJ Blokker T Montalvan B Girard O On the use of the repeated-sprint training in hypoxia in tennis. Front Physiol. (2020) 11:588821. 10.3389/fphys.2020.588821 33424620 PMC7793694

[24] Racinais S Alonso JM Coutts AJ Flouris AD Girard O González-Alonso J Consensus recommendations on training and competing in the heat. Br J Sports Med. (2015) 49(18):1164–73. 10.1136/bjsports-2015-094915 26069301 PMC4602249

[25] Pandolf KB. Time course of heat acclimation and its decay. Int J Sports Med. (1998) 19(Suppl 2):S157–160. 10.1055/s-2007-971985 9694426

[26] Shapiro Y Moran D Epstein Y. Acclimatization strategies–preparing for exercise in the heat. Int J Sports Med. (1998) 19(Suppl 2):S161–163. 10.1055/s-2007-971986 9694427

[27] Brechbuhl C Girard O Millet GP Schmitt L. Technical alterations during an incremental field test in elite male tennis players. Med Sci Sports Exerc. (2017) 49(9):1917–26. 10.1249/MSS.0000000000001303 28422770

[28] Brechbuhl C Girard O Millet G Schmitt L. Stress test specific to tennis (test): case study of an elite player. ITF Coach Sport Sci Rev. (2016) 24(70):27–30. 10.52383/itfcoaching.v24i70.217

[29] Ratel S Blazevich AJ. Are prepubertal children metabolically comparable to well-trained adult endurance athletes? Sports Med. (2017) 47(8):1477–85. 10.1007/s40279-016-0671-1 28044282

[30] Ratel S. Préparation physique du jeune sportif—Le guide scientifique et pratique. Amphora (Editions). (2018).

[31] Mirwald RL Baxter-Jones AD Bailey DA Beunen GP. An assessment of maturity from anthropometric measurements. Med Sci Sports Exerc. (2002) 34(4):689. 10.1097/00005768-200204000-00020 11932580

[32] Lloyd RS Oliver JL. The youth physical development model: a new approach to long-term athletic development. Strength Cond J. (2012) 34(3):61–72. 10.1519/SSC.0b013e31825760ea

[33] Sinkovic F Novak D Foretic N Zemková E. The effects of biological age on speed-explosive properties in young tennis players. J Funct Morphol Kinesiol. (2023) 8(2):48. 10.3390/jfmk8020048 37092380 PMC10123721

[34] Warren M Lininger M Chimera N Smith C. Utility of FMS to understand injury incidence in sports: current perspectives. Open Access J Sports Med. (2018) 9:171–82. 10.2147/OAJSM.S149139 30233259 PMC6135213

[35] Bonazza NA Smuin D Onks CA Silvis ML Dhawan A. Reliability, validity, and injury predictive value of the functional movement screen: a systematic review and meta-analysis. Am J Sports Med. (2017) 45(3):725–32. 10.1177/0363546516641937 27159297

[36] Moran RW Schneiders AG Mason J Sullivan SJ. Do functional movement screen (FMS) composite scores predict subsequent injury? A systematic review with meta-analysis. Br J Sports Med. (2017) 51(23):1661–9. 10.1136/bjsports-2016-096938 28360142

[37] Cook G Burton L Hoogenboom BJ Voight M. Functional movement screening: the ose of fundamental movements as an assessment of function—part 1. Int J Sports Phys Ther. (2014) 9(3):396–409. 24944860 PMC4060319

[38] Schmitt L Regnard J Parmentier A Mauny F Mourot L Coulmy N Typology of “fatigue” by heart rate variability analysis in elite nordic-skiers. Int J Sports Med. (2015) 36(12):999–1007. 10.1055/s-0035-1548885 26252552

[39] Stanley J Peake JM Buchheit M. Cardiac parasympathetic reactivation following exercise: implications for training prescription. Sports Med. (2013) 43(12):1259–77. 10.1007/s40279-013-0083-4 23912805

[40] Laborde S Mosley E Thayer JF. Heart rate variability and cardiac vagal tone in psychophysiological research—recommendations for experiment planning, data analysis, and data reporting. Front Psychol. (2017) 8:213. 10.3389/fpsyg.2017.00213 28265249 PMC5316555

[41] Schaffarczyk M Rogers B Reer R Gronwald T. Validity of the polar H10 sensor for heart rate variability analysis during resting state and incremental exercise in recreational men and women. Sensors. (2022) 22(17):6536. 10.3390/s22176536 36081005 PMC9459793

[42] Pomeranz B Macaulay RJ Caudill MA Kutz I Adam D Gordon D Assessment of autonomic function in humans by heart rate spectral analysis. Am J Physiol-Heart Circ Physiol. (1985) 248(1):H151–3. 10.1152/ajpheart.1985.248.1.H151 3970172

[43] Kovacs MS Baker LB. Recovery interventions and strategies for improved tennis performance. Br J Sports Med. (2014) 48:18–21. 10.1136/bjsports-2013-093223 24668374 PMC3995240

[44] Close GL Sale C Baar K Bermon S. Nutrition for the prevention and treatment of injuries in track and field athletes. Int J Sport Nutr Exerc Metab. (2019) 29(2):189–97. 10.1123/ijsnem.2018-0290 30676133

[45] Beelen M Burke LM Gibala MJ van Loon L JC. Nutritional strategies to promote postexercise recovery. Int J Sport Nutr Exerc Metab. (2010) 20(6):515–32. 10.1123/ijsnem.20.6.515 21116024

[46] Burke LM Loucks AB Broad N. Energy and carbohydrate for training and recovery. J Sports Sci. (2006) 24(7):675–85. 10.1080/02640410500482602 16766497

[47] Maughan RJ Burke LM Dvorak J Larson-Meyer DE Peeling P Phillips SM IOC consensus statement: dietary supplements and the high-performance athlete. Br J Sports Med. (2018) 52(7):439–55. 10.1136/bjsports-2018-099027 29540367 PMC5867441

[48] Ranchordas MK Rogerson D Ruddock A Killer C. Nutrition for tennis: practical recommendations. J Sports Sci Med. (2013) 12:211–24. 24149799 PMC3761836

[49] National Sleep Foundation (NSF). Sleep foundation. How much sleep do we really need? (2009). Disponible à: https://www.sleepfoundation.org/how-sleep-works/how-much-sleep-do-we-really-need (Cité Août 19, 2023).

[50] Watson AM. Sleep and athletic performance. Curr Sports Med Rep. (2017) 16(6):413–8. 10.1249/JSR.0000000000000418 29135639

[51] Lever JR Murphy AP Duffield R Fullagar HHK. A combined sleep hygiene and mindfulness intervention to improve sleep and well-being during high-performance youth tennis tournaments. Int J Sports Physiol Perform. (2021) 16(2):250–8. 10.1123/ijspp.2019-1008 32781440

[52] Kirschen GW Jones JJ Hale L. The impact of sleep duration on performance among competitive athletes: a systematic literature review. Clin J Sport Med Off J Can Acad Sport Med. (2018) 30(5):503–12. 10.1097/JSM.0000000000000622 29944513

[53] Duffield R Murphy A Kellett A Reid M. Recovery from repeated on-court tennis sessions: combining cold-water immersion, compression, and sleep interventions. Int J Sports Physiol Perform. (2014) 9(2):273–82. 10.1123/ijspp.2012-0359 23799825

[54] Reyner LA Horne JA. Sleep restriction and serving accuracy in performance tennis players, and effects of caffeine. Physiol Behav. (2013) 120:93–6. 10.1016/j.physbeh.2013.07.002 23916998

[55] Adão Martins NR Annaheim S Spengler CM Rossi RM. Fatigue monitoring through wearables: a state-of-the-art review. Front Physiol. (2021) 12:790292. 10.3389/fphys.2021.790292 34975541 PMC8715033

